# Route to Measure Exact Parameters of Bio-Nanostructures Self-Assembly

**DOI:** 10.3390/biom14111388

**Published:** 2024-10-31

**Authors:** Mikhail Kryuchkov, Jana Valnohova, Vladimir L. Katanaev

**Affiliations:** 1 Department of Cell Physiology and Metabolism, Faculty of Medicine, University of Geneva, Rue Michel Servet 1, CH-1211 Geneva, Switzerland; jana.valnohova@unige.ch; 2 School of Chemistry and Pharmaceutical Engineering, Shandong First Medical University (Shandong Academy of Medical Sciences), Tai’an 271016, China

**Keywords:** Turing, reaction-diffusion, reaction rate, order, dispersion relation

## Abstract

Artificial bio-nanocoatings, primarily composed of proteins, offer a broad range of applications across various fields thanks to their unique properties. Proteins, as major components of these structures, enable a high degree of customization, such as mutations, conjugation with other molecules or nanoparticles, or the inclusion of an enzymatic activity. Their ability to self-assembly simplifies the production of bio-nanocoatings, making this process efficient and environment-friendly. Despite these advantages, a comprehensive understanding of the underlying self-assembly mechanism is lacking, and the reaction rates governing this process have not been characterized. In this article, we introduce a novel method to determine the key parameters describing the self-assembly mechanism of bio-nanostructures. For the first time, this approach enables an accurate calculation of the autocatalytic and self-inhibitory parameters controlling the process. Through mathematical modeling, our method enhances the understanding of how the protein-based nanocoatings form and opens new avenues for their application in nanotechnology and synthetic biology. Improved control over the self-assembly processes may enable the development of nanomaterials optimized for specific functions, such as drug delivery, biosensing, and bioactive surface fabrication.

## 1. Introduction

**Figure 1:**
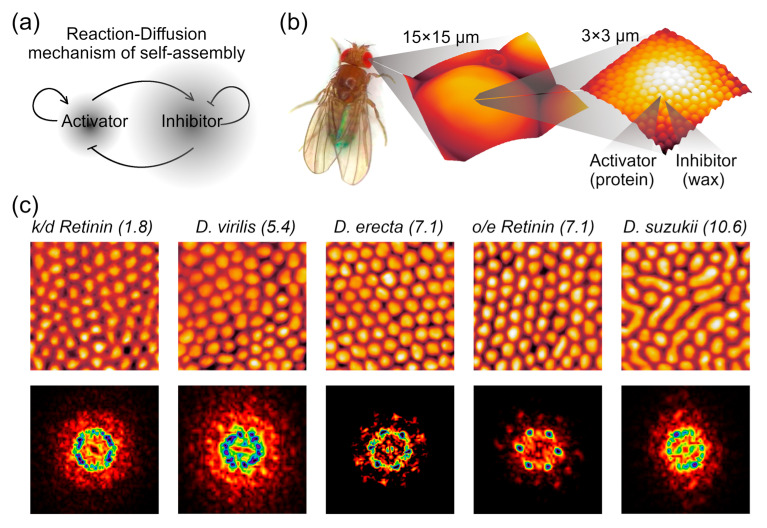
Bio-nanostructures formed by RD mechanism. (**a**) The Turing RD model involves a slowly diffusing autocatalytic activator, which stimulates its own production while also activating a fast-diffusing self-inhibiting inhibitor. (**b**) Nanoprotrusions cover each eyelet of Drosophila. These nanostructures consist of a protein (activator) and wax-like molecules (inhibitor); (**c**) Representative AFM scans of fruit fly corneas, positioned according to the relative protein content. *k*/*d* is knock-down and *o*/*e* is overexpression. Each scan is 2 × 2 µm; height is not to scale. In parenthesis above each AFM image, the levels of Retinin (percentage of Retinin to the total protein load in corneal preparations, taken from [7]) are provided. Fourier transformation (FFT) of the corresponding scans of the dimension 30 × 30 µm^−1^ is provided in the bottom line.

In 1952, Alan Turing published a theory on the self-assembly of patterns in the context of morphogenesis [1]. According to his model, two chemicals (an activator and an inhibitor) can cause reaction instability if they interact with each other in a specific way and diffuse through the medium at different rates. This model was called the Reaction-Diffusion (RD) mechanism (Figure 1a). Over the following decades, numerous examples of RD patterning have been discovered in living organisms, including lizard skin coloration [2,3], finger formation [4,5], left–right asymmetry in vertebrates [6], and more. These processes typically involve cell-to-cell communication with thousands of interacting molecules. However, a system involving only two chemicals has been found to create nanoscale protrusions on the cuticles of insects. These nanostructures are composed of a unique family of Retinin-like proteins, which act as Turing activators, and wax-like molecules functioning as the inhibitors (Figure 1b). Thanks to their self-assembly and simple composition, these nanostructured patterns have been successfully recreated in vitro [7]. The simplicity and mild conditions of their formation make them suitable to be applied on delicate surfaces, such as plastics and living tissues. Additionally, the possibility of protein modifications opens the door to creating nanostructures with enzymatic activities or enabling the metal nanoparticle conjugation thus granting diverse functionalities to the nanocoatings [8,9,10,11,12,13]. For example, silver nanoclusters can self-assemble under the control of specific peptides, producing temperature sensors or sensors for diverse small molecules [14]. Silver and copper nanoparticle-conjugated nanocoatings have shown antibacterial and antiviral properties while remaining non-cytotoxic due to the formation of a protein corona around the nanoparticles [15]. More complex structures requiring multiple activators have been observed in organisms like springtails, whip spiders, or whiteflies and could potentially be used to develop omniphobic or anti-contamination surfaces [16,17,18]. Furthermore, such protein-based modified assemblies can be used as tools to visualize or control processes inside living cells [19,20]. These advances pave the way for a wide range of applications, from sensors to cosmetics.

The diverse applications of these systems demand varying properties of the Retinin-like proteins that drive the self-assembly and nano-pattern formation. Despite the progress in the discovery and characterization of Retinin-like proteins [15], only their diffusion coefficients have been directly measured [21]. Their reaction parameters have remained unknown, and the methodology for determining these characteristics has been lacking. To date, only an approximate equation operating with these parameters has been developed [22,23,24]. Yet without knowing the precise parameters of the system, its mathematical investigation remains limited. Here, we provide a simple method that allows one to decrease the number of variables in the RD system of non-linear differential equations, which, in the next step, will allow one to make very precise simulations of pattern formation in two and three dimensions. A comprehensive method that fully describes the behavior of the patterning proteins could greatly enhance our understanding of the connection between the protein sequence and its ability to generate Turing instability with pre-selected parameters, a key factor for advancing toward applied nanotechnology and synthetic biology [25].

## 2. Materials and Methods

The *D. melanogaster* lines and other *Drosophila* species used in this study are described in the previous article; their corneal samples were prepared following the same protocol [7].

Artificial nanocoatings were generated using the method described in [7], with 0.006 mM *D. melanogaster* Retinin and 1.6 mM lanolin wax, with the coating process repeated twice.

Atomic force microscopy (AFM) of the *Drosophila* species was conducted by the NTegra-Prima microscope (NT-MDT, Moscow, Russia) and NSG 11 long (NT-MDT, Moscow, Russia) as a cantilever in contact mode. The topographical data for the artificial nanocoatings were acquired in contact mode with an XE-100 (Park Systems, Suwon, South Korea) microscope at the micro and nanotechnology facility (HES-SO, Geneva, Switzerland). The Gwyddion software (Department of Nanometrology, Czech Metrology Institute, Brno, Czech Republic) was used for visualization, quantification, and analysis.

Simulation 2D patterns were generated using the software RDsimJ.jar [26] with parameter values listed in Table S1.

For the Lyapunov exponent plot, images were created with the Desmos Graphing Calculator, which was used with permission from Desmos Studio PBC. The parameter values are listed in Table S1. The Mathematica, Version 13.3, from Wolfram Research, Inc. (Champaign, IL, USA), was used to calculate the reaction rate parameters. Statistical analysis was performed using GraphPad Prism Version 10.0.3.

## 3. Results

### 3.1. Order as a Function of the Activator’s Level

Corneal nanocoatings from various *Drosophila* species and *D. melanogaster* mutants were investigated using AFM. Representative results are shown in Figure 1c. These images reveal significant variation in nanostructural organization across species and genotypes: certain samples exhibit ordered nanostructures, while others display a rather random distribution of protrusions.

One commonly used technique for evaluating the degree of order and packing density is the Fourier transformation. The reflexes in the Fourier transforms provide valuable information about the degree of crystallinity in the packing [27]. Moreover, concentric circles indicate the uniformity of the shapes and dimensions of the individual nanostructures. The Fourier transformation of the AFM scans shows that certain samples exhibit clear hexagonal packing (Figure 1c, bottom row).

**Figure 2:**
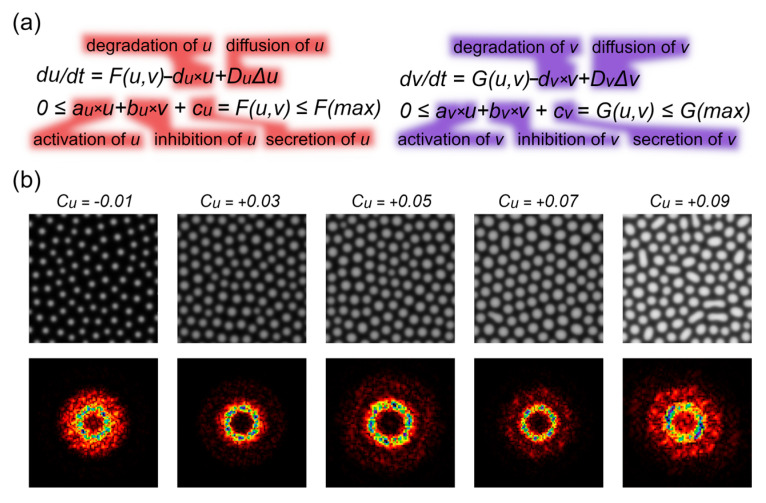
Patterns simulated by using RD equations. (**a**) Equations used for the simulation of the behavior of an activator (u) and an inhibitor (v), with a legend for each equation component. (**b**) Representative patterns generated at various activator secretion levels (c_u_). The corresponding FFTs of these scans are provided in the bottom row. Fourier transformation (FFT) is provided in the bottom line.

The key variable that changes across these samples is the concentration of Retinin-like protein. We simulated the pattern formation using known parameters to explore whether activator protein levels affect the nanostructural order. By slightly increasing the activator secretion parameter, we observed a progressive increase in the structural order, which then reversed as the nanostructures began to merge (Figure 2).

A Fourier analysis of the simulated patterns (Figure 2b, bottom row) reveals that at low activator levels, the order is rather low, which is evident as a single, relatively homogeneous circle of reflexes. As the activator concentration increases (c_u_ = +0.05), a second circle of reflexes emerges, while the first circle starts to exhibit hexagonal heterogeneity, indicative of an increase in order towards the hexagonal packing. With a further increase in the activator level (c_u_ = +0.07), both the first and second sets of reflexes display the heterogeneous and hexagonal arrangement, demonstrating a near-perfect hexagonal packing of the individual nanostructures and a state close to the maximal order. However, with a further increase in the activator level, reflexes merge into a shapeless cloud, resulting from a significant loss of order due to the fusion of individual protrusions into ridges.

**Figure 3:**
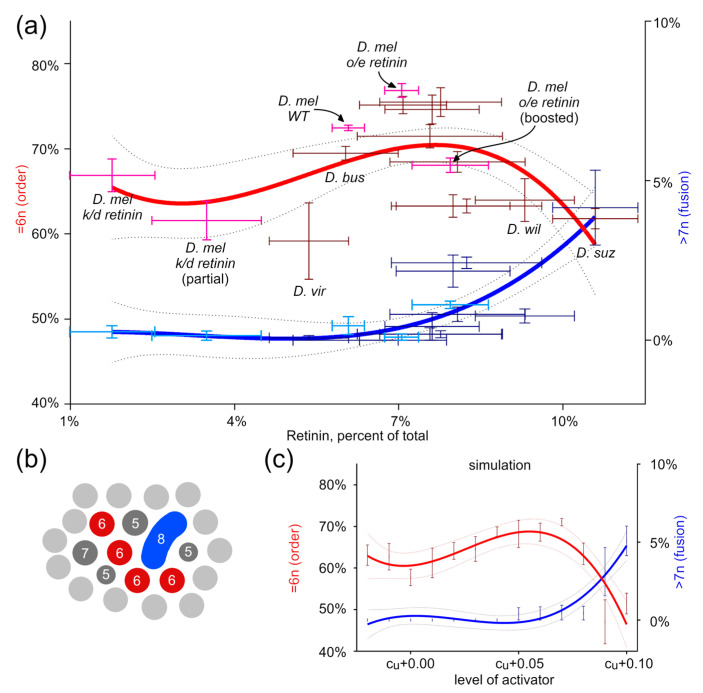
Relationship between hexagonal packing in corneal nanostructures and Turing activator levels. (**a**) Correlation between the activator (Retinin) levels and the degree of order in the packing of individual nanostructures, or the degree of their fusion. The degree of order was determined as the percentage of individual nanostructures with six neighbors. The degree of fusion of nanostructures is determined by the percentage of individual protrusions with >7 neighbors. Data is presented as mean ± SEM for both dimensions. Pink and light-blue colors highlight *D. melanogaster*, wild-type, and mutants; other *Drosophila* species are given brown and blue colors. Nonlinear regressions are plotted with CI 95% shown as dotted lines. (**b**) Illustration of the method of counting the number of neighbors for an individual protrusion. (**c**) The same correlation as in (**a**) was obtained after the Turing simulation. The level of activator was set manually to run the simulation; each point represents three different simulations, and data is provided as mean ± SEM. Nonlinear regressions are plotted with CI 95% shown as dotted lines. Note the remarkable similarity between the experimental data using the Retinin levels (**a**) and the Turing’s activator levels (**c**).

In the next step, we analyzed the order by the percentage of protrusions that undergo hexagonal packing. Simulation data show that the order increases with the higher activator levels. We compared these results with the corneal nanocoatings from the biological samples to confirm that this behavior is not merely a simulation artifact. *D. melanogaster,* a convenient model for gene expression studies, allowed us to downregulate and overexpress Retinin, taking advantage of the natural differences between different *Drosophila* species [7]. We plotted the order and the fusion levels against the relative Retinin-like protein levels. With these data, we see similar trends (Figure 3).

### 3.2. Spread of the Lyapunov Exponent’s Positive Part Is Minimized for Ordered Structures

The Lyapunov exponents (*λ*) are derived from the linearized form of the RD equations around the steady state, and obtaining the exponents permits the analysis of how small perturbations in the system’s parameters affect the whole system. The stability of the system is assessed by examining the linearized equations’ eigenvalues, which correspond to the Lyapunov exponents [28,29,30].

In our analysis, the RD equations are modified by the cubic coupling [7,31] of the secretion rates:

(1) $$\left\{\begin{array}{c} \frac{\partial u}{\partial t}=D_{u}\Delta u+a_{u}u+b_{u}b+c_{u}u(u-c_{u})\;\\ \frac{\partial v}{\partial t}=D_{v}\Delta v+a_{v}u+b_{v}v+c_{v}v(v-c_{v}) \end{array}\right.$$

Knowing, that

(2) $$Det(A-\lambda I-k^{2}D)=0\;,\;\mathrm{and}\;A=J{\left(\begin{array}{cc} a_{u}-2c_{u}u+{c_{u}}^{2} & b_{u}\\ a_{v} & b_{v}-2c_{v}v+{c_{v}}^{2} \end{array}\right)}_{u_{0}{;\;v}_{0}}$$

we can find *λ* from the quadratic equation:

(3) $$\left(a_{u}+{m\left(c_{u}-l\right)}^{2}-\lambda -D_{u}k^{2}\right)\left(b_{v}+{n\left(c_{v}-p\right)}^{2}-\lambda -D_{v}k^{2}\right)-a_{v}b_{u}=0\;$$

where *m*, *l*, *n*, and *p* are unknown coefficients. This equation will be further used to calculate all Lyapunov exponents, with the parameters listed in Table S1.

**Figure 4:**
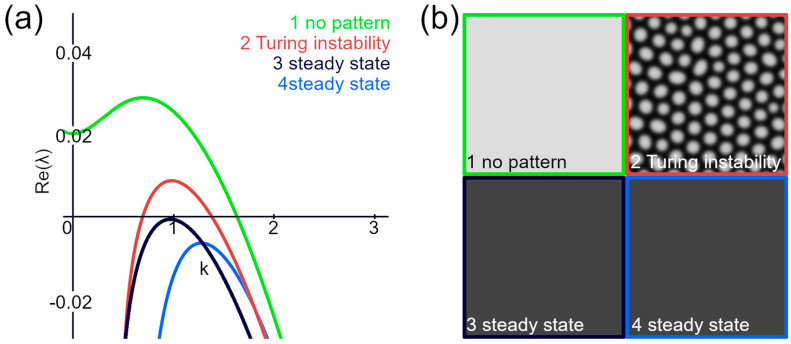
Lyapunov exponent and Turing instability. (**a**) Lyapunov exponent is plotted for various parameters that either produce or do not produce a Turing pattern. (**b**) Results of the RD simulation using the same parameters as in (**a**).

The Lyapunov exponent provides a measure of the rate of separation of infinitesimally close trajectories in dynamic systems. In the context of RD equations, it characterizes the stability of systems around the steady state. Specific conditions for the Turing instability can be formulated in terms of the Lyapunov exponents. For instance, the Turing instability is confirmed if the maximal real part of the Lyapunov exponent has a negative value at the origin and throughout most of its length, with only a part being positive (Figure 4a,b).

**Figure 5:**
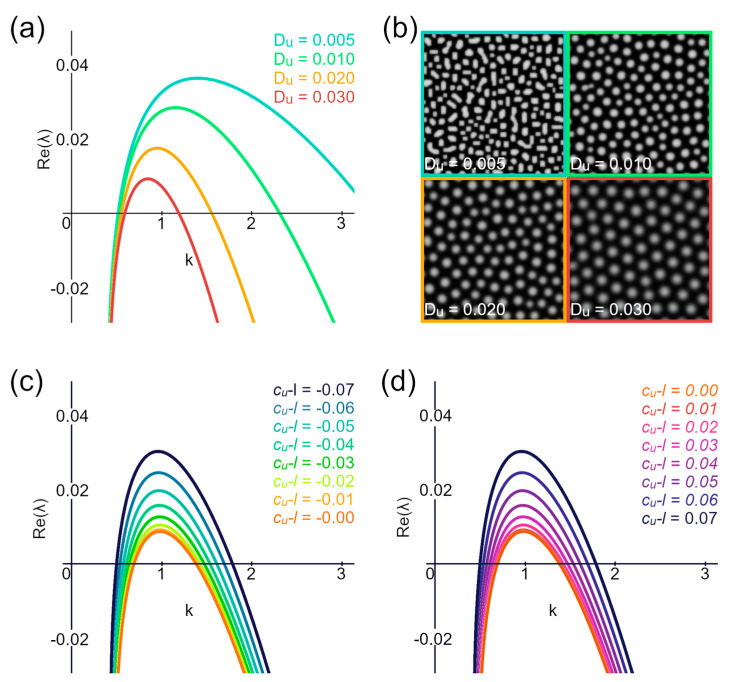
Lyapunov exponent and its connection to hexagonal packaging. (**a**) Lyapunov exponents for varying activator diffusion coefficients (*D_u_*). (**b**) Results of simulation using the parameters from (**a**). (**c**,**d**) The positive part of the Lyapunov exponent shows minimal spread when the equation component, related to secretion (*c*_u_-l), equals zero.

Our intuition might suggest that pattern packing involves pushing individual structures apart from each other. However, the real reason for the tight packaging lies in the existence of only a narrow window of distances from a given structure where a new structure can exist. Knowing that *k* = 2*π*/*L*, where *k* is the wavenumber component, and *L* is the domain length [22] and that the time evolution of a specific wavenumber component is described as follows:

(4) $${{\vec{u}}_{0}e}^{\lambda t}\sin\left(kx\right)$$

we can calculate that a particular wavenumber component will either grow or decay depending on the sign of *λ* [26]. Increasing the range of positive *λ* values raises the uncertainty of the localization of a pattern unit appearing, leading to a loss of order. This effect can be demonstrated by an example of the activator diffusion coefficient (*D_u_*) changes, which have been shown to affect the order of structures [32]. As the activator diffusion coefficient increases, the range of positive *λ* values decreases while the hexagonal packaging and pattern order improve (Figure 5a,b).

Varying the activator expression rate (*c_u_*) reveals that the lower range of positive *λ* values occurs when the equation *m*(*c_u_-l*) part equals zero (Figure 5c,d). At the same time, simulations show that the most ordered structures are obtained when *c_u_* equals 0.07. Based on this data, we suggest that, for Equation (3), the secretion part and all unknown coefficients can be disregarded when aiming for the most ordered structures.

### 3.3. Reaction Rate Determination Method

**Figure 6:**
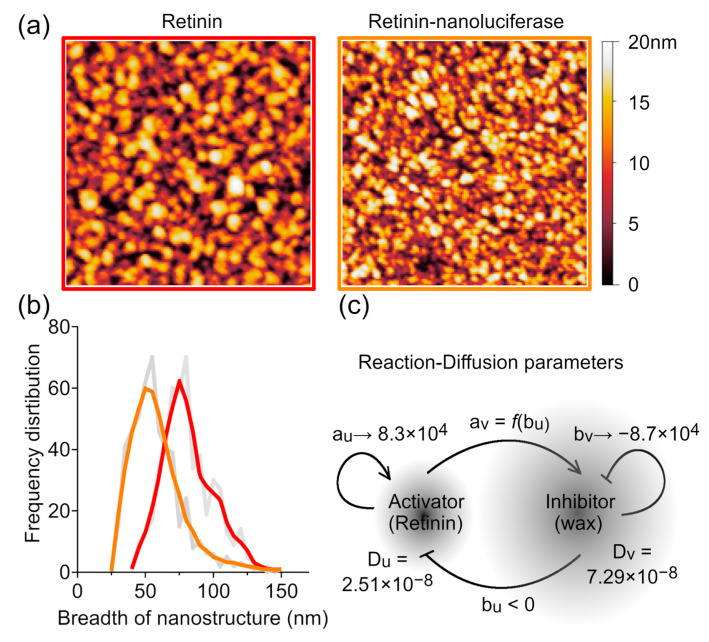
Reaction rate determination method. (**a**) AFM scans of Retinin and Retinin-nanoluciferase-based artificial nanocoatings with the maximal level of order. Each image is 1 × 1 μm. (**b**) Distribution of frequencies of the nanostructure breadths (grey lines), fitted with the LOWESS curves: Retinin—red, Retinin-nanoluciferase—orange. *n* = 500. (**c**) Turing RD model with parameters defined for the Retinin protein.

The Lyapunov exponent derived from Equation (3) can be used to analyze artificial nanocoatings. When nanocoatings are created on a surface using wax and protein solutions with known concentrations, the model is much simpler than describing the tightly regulated secretion from specialized cells [33,34]. Additionally, it does not require the three-dimensional analysis with diffusion of components through the *z* axis, as is the case for the insect corneal nanostructures [7,21]. By adjusting the concentrations of Retinin-like protein and wax in the initial solution, it is possible to identify parameters that lead to the formation of the most ordered nanostructures. For the Retinin protein, such concentration screening of parameters has been performed in our previous article [7]. These structures must have a distribution of nanostructure breadths that corresponds to the range of the positive part of the Lyapunov exponent, with the major breadth reflected in the maximal Lyapunov exponent *λ_max_* (Figure 6a,b).

Therefore, we can apply these parameters to Equation (3) by transforming *k* = 2*π*/*L*. When *λ* = *λ_max_* from [22]:

(5) $$k^{2}=\frac{-D_{u}D_{V}\left(a_{u}-b_{v}\right)+(D_{u}+D_{V})\sqrt[{2}]{-D_{u}D_{v}b_{u}a_{v}}}{D_{u}D_{v}(D_{v}-D_{u})}$$

Using the equations for *λ_o_* and *λ_max_* and applying Turing instability parameters restriction set [22]:

(6) $$\;{a_{u}+b_{v}<0;\;a}_{u}b_{v}-b_{u}a_{v}>0;\;D_{v}a_{u}+D_{u}b_{v}>0;\;{{(D}_{v}a_{u}+D_{u}b_{v})}^{2}-4D_{u}D_{v}\left(a_{u}b_{v}-b_{u}a_{v}\right)>0\;$$

from the known diffusion parameters [21], we can calculate the exact reaction rates for *a_u_* and *b_v_* parameters and the relationship between *a_v_* and *b_u_* parameters. For Retinin, *a_u_* is equal to 8.3 × 10^4^ ± 0.7 × 10^4^ taking into account the uncertainty of the different diffusion coefficients, *b_v_* ≈ −8.7 × 10^4^ ± 0.4 × 10^4^, and the following function describes the relation between the *a_v_* and *b_u_*:

(7) $$a_{v}\approx -\frac{(9\pm 3)\cdot 10^{9}}{b_{u}}\;$$

that converge at 9.5 × 10^4^ for *a_v_* and *b_u_* (Figure 6c). These findings are consistent with our previous assumptions and approximations [7,32].

However, it was shown that Retinin fusion with nanoluciferase does not affect its interaction with the inhibitor but changes its diffusion coefficient [21]. Using this construct, we re-measured the reaction rate for Retinin by using different diffusion rates and nanostructure sizes (Figure 6b). The reaction rates for Retinin-nanoluciferase showed an overlap with the unmodified Retinin characteristics: *a_u_* ≈ 8.2 × 10^4^ ± 0.7 × 10^4^, *b_v_* ≈ −8.6 × 10^4^ ± 0.4 × 10^4^, and the relation between *a_v_* and *b_u_*:

(8) $$a_{v}\approx -\frac{(8\pm 3)\cdot 10^{9}}{b_{u}}\;$$

that converge at 8.9 × 10^4^ for *a_v_* and *b_u_*.

## 4. Discussion

**Figure 7:**
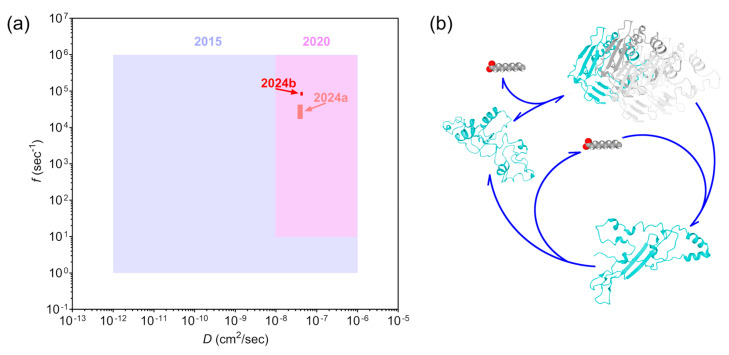
Progress in the bio-nanostructures self-assembly research. (**a**) Year-to-year changes in RD parameters identification. *D* represents the joint diffusion coefficient; *f* denotes the joint reaction rate. (**b**) One of the possible models of protein-wax interaction includes Retinin folding and supramolecular complex assembly (different molecules marked by colors), instigated by the interaction with wax and its disassembly upon further wax binding. Retinin structures predicted by I-TASSER (Available online: https://zhanglab.ccmb.med.umich.edu/I-TASSER, accessed on 29 October 2024).

Previously, in 2015, the mechanism of nanostructure self-assembly was described as the formation of nanopatterns in a colloidal-like system with reduced diffusion parameters. The underlying mechanisms of the patterning involved aggregation, polymerization, and disassembly of the interacting molecules of unknown nature. For this model, the reaction rate was estimated to be in a range from 1 to 1 million s^−1^, with diffusion of components from 10^−12^ to 10^−6^ cm^2^/s [32]. Then, in 2020, the identification of the activator and the inhibitor led to the revised parameter ranges of 10–10^6^ s^−1^ and 10^−8^–10^−6^ cm^2^/s [7]. By 2024, the exact diffusion coefficients were directly measured [21], and other parameters were approximately calculated (Figure 7a (2024a)).

The new method for identifying RD parameters proposed in this article allowed us to specify each reaction rate and diffusion coefficient (Figure 7a (2024b)). The difference between the earlier and the now-calculated reaction rates can be attributed to the fact that the equation used in the previous article was intended to provide a rough understanding of these parameters with an accuracy of one order of magnitude. Although we have not re-measured the diffusion coefficient in this work, the measured parameters and equations produced results only within a part of the diffusion coefficient range for both Retinin and Retinin-nanoluciferase constructs. In the first case, only higher values were compatible, whereas, for the fused protein, only lower values were compatible. Based on these results, we refined the previously calculated values for both reaction and diffusion.

The existence of the *m*(*c_u_-l*) part in the equation suggests that it is not a Retinin-like protein that plays an activator role, but rather a Retinin-wax complex [35]. We propose that the interaction of these molecules is similar to microtubule assembly [36,37,38] in three dimensions, the difference being that the disassembly does not require the processing of ATP but instead relies on the binding of additional wax molecules (Figure 7b). The assembled molecules recruit single molecules and activate their folding, leading to the self-activation of the activator. At the same time, they provide the valency for the binding of wax molecules, which changes their conformation and results in partial disassembly of the complex. Moreover, this interaction plays the role of an inhibitor, as it is necessary for the dismantling of the nanostructure. Since the interaction depends on the wax availability, we can estimate that the diffusion coefficients of wax and the wax-binding valences are equal. The actual interaction mechanism can be much more complex, as it was discovered for the Belousov−Zhabotinsky reaction [39,40], and requires further research with precise kinetics measurements and curve-fitting. Considerable progress was made in the studies of protein aggregation last years [41,42], for example using the iso- and aniso-tropic Thioflavin T fluorescence kinetics measurements [43].

Applying the complete set of morphogen concentrations can extend our approach to identifying secretion and primary interaction constants, allowing us to obtain numeric solutions for the *a_v_* and *b_u_* parameters. This is feasible due to subtle variations in the Lyapunov exponent behavior near the steady state and the non-Turing mode [44]. Another promising direction is using *λ_max_* to determine the time of self-assembly since an increase in *λ_max_* is predicted to reduce the time needed for pattern appearance [22].

As this method provides the exact parameters for the simplified model of interactions, it can be applied to other Turing patterning processes with known coefficients of mobility or signal transduction, like prey-predator interaction [45,46] or investigation of neurons excitation [47,48]. However, it requires the existence of the Turing instability with its maximal order.

## 5. Conclusions

In this article, we provide a new simple method to investigate the parameters underlying the RD mechanism. This method requires only the knowledge of the diffusion parameters and an image of the tightly packed nipple-like pattern in a two-dimensional space. Despite the fact that this method can be used for any Turing pattern [49,50], it is of particular interest for protein-based self-assemblies.

Identification of reaction parameters enhances the understanding of Retinin-like proteins, enabling the connection of the proteins’ sequences to their self-assembly properties. Varying these parameters by protein modifications, or by shuffling protein domains, one can obtain a range of possible topographies, interactions, and instability areas. Retinin-like protein modification opens new routes for various applications, contributing to the advancement of self-assembling metamaterials [51,52,53]. Even more importantly, it allows one to predict the behavior of new proteins, identify new Retinin-like proteins in genomes, or reverse engineer protein sequences based on the desired physical properties of the ultimate bio-inspired nanocoatings.

## Data Availability

All data generated or analyzed during this study are included in this published article and its Supplementary Information files.

## Supplementary Materials

The following supporting information can be downloaded at: https://www.mdpi.com/article/10.3390/biom14111388/s1, Table S1: Parameters, used for simulations and Lyapunov exponent calculation.
