# Supplementary material for: Route to Measure Exact Parameters of Bio-Nanostructures Self-Assembly
Source: Biomolecules. 2024 Oct 31;14(11):1388. doi: 10.3390/biom14111388 (PMC11592367; doi:10.3390/biom14111388)
Supplement: Supplementary file 1 [file biomolecules-14-01388-s001.zip › biomolecules-3277085-supplementary.pdf]

biomolecules

MDPI

Supplementary Materials

Table S1. Parameters, used for simulations and Lyapunov exponent calculation.

	d_u	D_u	a_u	b_u	c_u	F_{max}	d_v	D_v	a_v	b_v	c_v	G_{max}	m	l
Figure 2b	0.03	0.02	0.08	-0.08	from -0.01 to 0.09	0.2	0.08	0.5	0.15	0.0	-0.15	0.5	—	—
Figure 3b	0.03	0.02	0.08	-0.08	from -0.02 to 0.12	0.2	0.08	0.5	0.15	0.0	-0.15	0.5	—	—
Figure 4a	—	0.02	0.05	-0.08	0.07	—	—	0.5	0.15	-0.3	0.0	—	3.1	0.07
	—	0.02	0.05	-0.08	0.07	—	—	0.5	0.15	-0.08	0.0	—	3.1	0.07
	—	0.02	0.04	-0.08	0.07	—	—	0.5	0.15	-0.08	0.0	—	3.1	0.07
	—	0.02	0.05	-0.2	0.07	—	—	0.5	0.15	-0.08	0.0	—	3.1	0.07
Figure 4b	0.03	0.02	0.08	-0.08	0.07	0.2	0.08	0.5	0.15	-0.22	-0.15	0.5	—	—
	0.03	0.02	0.08	-0.08	0.07	0.2	0.08	0.5	0.15	0.0	-0.15	0.5	—	—
	0.03	0.02	0.07	-0.08	0.07	0.2	0.08	0.5	0.15	0.0	-0.15	0.5	—	—
	0.03	0.02	0.08	-0.2	0.07	0.2	0.08	0.5	0.15	0.0	-0.15	0.5	—	—
Figure 5a	—	from 0.005 to 0.03	0.08	-0.08	0.07	—	—	0.5	0.15	-0.08	0.0	—	3.1	0.07
Figure 5b	0.03	from 0.005 to 0.03	0.08	-0.08	0.07	0.2	0.08	0.5	0.15	0.0	-0.15	0.5	—	—
Figure 5c,d	—	0.02	0.05	-0.08	from 0.0 to 0.14	—	—	0.5	0.15	-0.08	0.0	—	3.1	0.07

Supplementary note 1. Wolfram Matematica script for the parameters identification:

```
ClearAll[x, y, z, w, k, l, m, A, B]
k = 188246.2*2*Pi;
l = 365682.4*2*Pi;
m = 66893.2*2*Pi;
A = 0.0000000134;
B = 0.0000000729;
Solve [(-A*B*(x - w) + (A + B)*Sqrt[-A*B*z*y])/(A*B*(B - A)) - k^2 ==
   0 && 1/2*(Sqrt[(-x - w + A*l^2 + B*l^2)^2 -
        4*(x*w - x*B*l^2 - y*z - w*A*l^2 + A*B*l^4)] + x + w - A*l^2 -
       B*l^2) == 0 &&
  1/2*(Sqrt[(-x - w + A*m^2 + B*m^2)^2 -
        4*(x*w - x*B*m^2 - y*z - w*A*m^2 + A*B*m^4)] + x + w - A*m^2 -
       B*m^2) == 0 && x > 0 && y < 0 && z > 0 && w < 0 && x + w < 0 &&
   x*w - y*z > 0 && (B*x + A*w)^2 - 4*A*B*(x*w - y*z) > 0 &&
  B*x + A*w > 0, {x, y, z, w}]
```
